# Molecular profile of liquid biopsies: next generation biomarkers to improve lung cancer treatment

**DOI:** 10.3332/ecancer.2015.598

**Published:** 2015-11-24

**Authors:** Fabrizio Bianchi

**Affiliations:** Molecular Medicine Program, European Institute of Oncology, Milan 20141, Italy

**Keywords:** biomarkers, circulating biomarkers, circulating tumour DNA, lung cancer, microRNA

## Abstract

Molecular profiling of liquid biopsies is now emerging as pivotal for cancer biomarker discovery. The low-invasive nature of the approach used for collecting biospecimens (i.e. blood, urine, saliva, etc.) may allow a widespread application of novel molecular diagnostics based on liquid biopsies. This is relevant, for example, in cancer screening programmes where it is essential to reduce costs and the complexity of screening tests in order to increase study compliance and effectiveness. Here, I discuss recent advances in biomarkers for the early cancer detection and prediction of chemotherapy response based on the molecular profiling of liquid biopsies.

## Introduction

Despite significant improvements in the detection and treatment of cancer made in recent decades, which have had a positive impact on the overall survival of cancer patients [1], the prognosis of patients with advanced cancer (i.e. with regional or distant metastases) remains poor [1]. Lung cancer offers a prototypical example, while patients diagnosed with early-stage lung tumours (stage I–II) have a 5-year survival rate of ~40–50% [1], and up to ~90% for computerised tomography (CT)-detected stage-I tumour [2], the 5-year relative survival rate drops to 4–26% when the cancer is diagnosed at an advanced stage (~85% of all cases) [1]. The unfavourable prognosis of patients with advanced lung cancer is due to the metastatic spread of cancer cells to the regional (lymph nodes) or distant organs, which cannot be completely eradicated by surgery or multimodal therapy. In some cases, neoadjuvant chemotherapy (given before surgery) helps to reduce tumour size and to eradicate micrometastases in order to facilitate surgery in patients with stage-IIIA lung cancer [3]. However, patients treated with conventional chemotherapy (i.e. platinum based) frequently develop resistance to therapy due to the selection of molecular alterations in tumour cells, which make them resistant to most of the available chemotherapeutic agents [4]. For this reason, several investigators now focus their studies on the identification of cancer gene mutations, aimed at characterizing the molecular mechanisms that underlie chemoresistance, which may help to identify more effective therapeutic strategies for chemorefractant cancer [5].

With the advent of next-generation sequencing (NGS), which allows simultaneous sequencing of all coding genes in the human genome in a few days, it is now possible to deeply sequence hundreds of tumours to map the mutational landscape of cancer including mutations possibly involved in chemoresistance [6, 7]. Results from these studies are expected to change the way that we treated patients with cancer: from the concept of ‘one drug fits all’ to the concept of ‘personalised medicine’ using molecular targeted drugs. However, the successful treatment of neoplastic diseases is hindered by the complexity of the cancer genome which was confirmed by recent NGS studies [8–11]. The molecular complexity of cancer augments with disease progression, favoured by the acquisition of genomic instability in cancer [10, 12, 13]. In such scenario, new methods to anticipate diagnosis of cancer may increase the frequency of early diagnosis when surgery and chemotherapy are more effective. Recent results from large trials for the early detection of lung cancer showed a 20% reduction of mortality in individuals who underwent screening [14]. However, the number of screened individuals required to detect one lung cancer patient is still too high (~1 patient out of 100 screened individuals) and the high false-positive rate in CT screening (most of the lung nodules detected are benign, ~96%) represents a limit to the widespread applicability of screening programmes [14]. Thus, there is an urgent need to identify additional epidemiologic risk factors (other than age, smoking status) and cancer biomarkers to better identify the high-risk population, thus reducing the number of unnecessary exams.

## Circulating microRNA as new valuable biomarkers for lung cancer screening

The molecular profile of liquid biopsies (i.e. blood, urine, saliva) is an attractive field for cancer biomarkers discovery, both for the relative low invasivity of the procedure for collecting material and for the possibility to obtain multiple samples from the same patients at different times. Recently, the variety of circulating molecules analysable in blood samples has been further expanded with the discovery of circulating free-RNA and, in particular, of a specific class of small RNA called microRNAs that are remarkably stable in body fluids [15–17]. MicroRNAs (miRNAs) are short non-coding RNA molecules function as endogenous triggers of the mRNA interference pathway and are involved in the regulation of many cellular processes, including differentiation, proliferation and apoptosis [17, 18]. The expression of miRNAs is often deregulated in human tumours, both in a tissue- and in a cancer-specific manner [19]. Importantly, fluctuations of circulating miRNAs were shown to be associated with many malignant and non-malignant diseases, including lung cancer [15, 20, 21]. Contrary to circulating tumour-DNA (ct-DNA), where there are evidences that only a fraction of early stages tumours may release sufficient quantities of circulating DNA to be detected in the blood [22], circulating free-miRNAs (cf-miRNA) appear to be excellent candidates for bloodborne tumour markers for the early diagnosis of different tumours [23, 24].

In line with this, our group has recently characterised cf-miRNA signatures that correlate with the presence of asymptomatic, early stage, lung cancer [25, 26]. We initially analysed cf-miRNA in serum samples from 174 individuals enrolled in the COSMOS (Continuing Observation of SMOking Subjects) trial (ClinicalTrials.gov Identifier: NCT01248806 [27]). This trial assessed the efficacy of annual LDCT scans in detecting early-stage lung cancer in over 5000 high-risk individuals and validated simple and non-invasive guidelines for the diagnostic work up of screen-detected lung nodules [27, 28]. From the miRNA analysis, we derived a 34 serum cf-miRNA signature (Table 1) that can identify individuals with asymptomatic, early stage, lung cancer (AUC = 0.89, *p* < 0.0001) [25]. We used this signature to develop a diagnostic test, the miR-test (Table 1), which confirmed an AUC of 0.85, accuracy of 74.9%, sensitivity of 77.8% and specificity of 74.8% in an additional independent cohort of 1115 high-risk individuals from the COSMOS trial [29]. Another study, COSMOS 2, is ongoing and is screening by CT and miR-test ~10.000 high-risk subjects from eight different health institutions. Results of this study will confirm whether miR-test may better identify high-risk candidates for low-dose CT screening, thus reducing screening costs and augment feasibility. Initial results of the screening will be available in 2017.

Other cf-miRNA signatures for lung cancer early detection have recently been described [26, 30–33], though few of them have been validated in lung cancer screening studies. Almost half of these signatures were discovered by analysing serum samples, while the others were derived from plasma samples (Table 1). There are differences in both quantities and species of cf-miRNAs when analysing serum or plasma samples due to differences in the chemical composition and in the technical preparation of these two biological specimens [34, 35]. This should be taken into account when searching for overlapping cf-miRNA in the different studies or during validation of cf-miRNA biomarkers using external cohorts. However, Wozniak *et al*. recently described an analysis of multiple cf-miNA signatures derived both from serum (including our 34-miRNA signature) and plasma samples, in a case–control study of 100 lung cancer patients and 100 non-cancer controls, whose plasma samples were profiled by TaqMan Human MicroRNA Array [33]. Interestingly, our 34-miRNA serum signature performed well in this case–control cohort of plasma samples (AUC = 0.78) that suggests the existence of a core of cf-miRNAs which quantities and species do not vary significantly in the serum or plasma samples.

## Circulating tumour-DNA mutational screening can identify actionable mutations for lung cancer treatment

Pioneer studies demonstrated the presence of circulating free DNA (cf-DNA) in the blood and their potential applicability for screening pathological conditions including cancer [36, 37]. Subsequent studies further analysed cf-DNA and demonstrated that part of these DNA may be specifically released by cancer cells (circulating tumour DNA, ct-DNA) [38], thus corroborating the use of ct-DNA as a cancer biomarker [39–41]. An advantage of screening ct-DNA in the blood rather than circulating proteins (e.g. PSA, CA125, CEA) is that the former can be checked also for tumour-specific mutations which dramatically increase the specificity of a blood test for cancer diagnosis and monitoring. Importantly, ct-DNA screening was shown to be effective to determine EGFR mutation status in patients with metastatic lung cancer who acquired resistance to anti-EGFR tyrosine kinase inhibitors (TKI) [42–44]. For example, the acquisition of the EGFR T790M resistance mutation was shown to be traceable in ct-DNA before treatment with anti-EGFR TKI [45], which suggests that ct-DNA screening may allow the anticipation of third-generation anti-EGFR TKIs in the case of acquired resistance to first-line anti-EGFR TKI therapy (gefitinib, afatinib, erlotinib). However, the sensitivity of this assay across different tumour types and stages still represents a concern that should be addressed before clinical application [46].

In addition, the recent application of NGS in screening cf-DNA allowed for searching known or novel mutations in an ‘unsupervised’ manner, which eliminates the requirement of a prior knowledge of relevant mutations thus expanding the possibility of identifying novel mutations involved in cancer progression and chemoresistance [47]. An important application of NGS for screening ct-DNA was recently proposed by Newman *et al*, where a relatively inexpensive, yet highly sensitive, method called CAPP-Seq was developed to screen for 521 exons and 13 introns from a total of 139 mutated genes [22]. The method was sensitive enough to monitor treatment response in patients with lung cancer and to predict mutations possibly responsible for chemoresistance [22].

## Conclusion

I envision that the molecular analysis of liquid biopsies will become the gold standard for developing non-invasive tests for personalised medicine. Through a ‘simple’ blood test clinicians will be able to anticipate diagnosis of cancer thus augmenting the probability of curative treatments for patients with cancer. In addition, the possibility to collect multiple samples (even in the case of metastatic disease) from the same patient will help to better monitor the therapeutic response, and to aid oncologists to anticipate second line (molecularly targeted) chemotherapy, in the case of acquired chemotherapy resistance. It will also be essential to overcome the current technological limitations which impede the large-scale application of these molecular tests. Indeed, the extraction and analysis of circulating nucleic acids require expensive and complex equipment that is still available only in few genomics laboratories worldwide. Furthermore, the sensitivity of these molecular tests should be improved in order to augment the detection rate and avoid false-negative results that are detrimental for cancer screening programmes. The possibility to analyse circulating biomarkers directly in the blood without sample pre-processing and using point-of-care health diagnostic technologies will favour massive application of such molecular tests.

## Figures and Tables

**Table 1.** Published cf-miRNA signatures for lung cancer early detection.

Authors	PubMed ID	Number (miRNA)	AUC	Serum	Plasma	CT screening
Bianchi *et al*	21744498	34	0.89	–	–	–
Montani *et al*^§^	25794889	13	0.85	–	–	–
Boeri *et al*	21300873	13	0.88	–	–	–
Sozzi *et al*	24419137	24	–	–	–	–
Wozniak *et al*	25965386	24	0.78^*^	–	–	–
Nadal *et al*	26202143	4	0.99	–	–	–
Chen *et al*	21557218	10	0.97	–	–	–

Black cells indicate the kind of samples used to derive the signatures (plasma or serum) and whether the signatures were validated in lung cancer screening studies by low-dose computed tomography (CT screening). AUC, area under the curve of the receiver operating characteristic (ROC) curve.

§ miR-test composition: miR-92a-3p, miR-30b-5p, miR-191-5p, miR-484, miR-328-3p, miR-30c-5p, miR-374a-5p, let-7d-5p, miR-331-3p, miR-29a-3p, miR-148a-3p, miR-223-3p, miR-140-5p.

* Predicted performance when applied to independent samples.
